# Evaluation and Optimization of Thermoplastic Extrusion Parameters to Improve the Dimensional Accuracy of Additively Manufactured Parts Made of PETG, Recycled PETG, ASA, and Recycled ASA

**DOI:** 10.3390/polym18050573

**Published:** 2026-02-27

**Authors:** Dragos Gabriel Zisopol, Mihail Minescu, Dragos Valentin Iacob

**Affiliations:** 1 Mechanical Engineering Department, Petroleum-Gas University of Ploiesti, 100680 Ploiesti, Romania; 2 Department of Mechanical Engineering, Doctoral School, Petroleum-Gas University of Ploiesti, 100680 Ploiesti, Romania

**Keywords:** FFF parameters, experimental determinations, dimensional accuracy, process parameters optimization, recycled materials, sustainability

## Abstract

As additive manufacturing (AM) expands into high-end industries, ensuring both technical performance and dimensional accuracy remains a challenge. This paper addresses the challenge of integrating recycled materials into the field of plastic extrusion additive manufacturing technologies by conducting a study on the evaluation and optimization of thermoplastic extrusion parameters to improve the dimensional accuracy of additively manufactured parts from virgin and recycled polyethylene terephthalate glycol (PETG, rPETG) and acrylonitrile styrene acrylate (ASA), both in virgin and recycled form. To carry out the study, 180 three-point bending specimens were additively manufactured on the QIDI Q1 Pro 3D printer by thermoplastic extrusion of PETG, rPETG, ASA, rASA (45 specimens for each type of material), using the following variable parameters: layer height deposited in one pass L_h_ = (0.10–0.20) mm and filling percentage—I_d_ = (50–100)%. After manufacturing the specimens, the dimensional characteristics that will be determined by measurement were defined: L—length, WA—width A, HA—height A, WA’—width A’, and HA’—height A’. Dimensional accuracy was assessed through 900 measurements using a DeMeet 400 coordinate measuring machine and analyzing the arithmetic means, dispersions, and mean square deviations. The results of the study confirm the superior dimensional stability of virgin materials (18.77–20.04%) compared to recycled materials. The analysis demonstrates that by optimizing the process parameters, filaments from recycled materials (rPETG and rASA) can achieve acceptable precision, with average deviations of 0.25–0.78% from the nominal dimensions. The present study validates the use of rPETG and rASA as a viable alternative for applications that do not require critical tolerances.

## 1. Introduction

Fused filament fabrication (FFF) is among the most widely used additive manufacturing (AM) methods due to its accessibility and versatility and diversified range of materials [1,2]. In recent years, AM technologies have experienced major improvements represented by increased technical performance of equipment (speed, precision, and defect detection systems), improvements in slicer software used to set parameters and generate work instructions in G-Code format, but also improvements in material compatibility [3,4,5]. However, although the equipment is high-performance and the materials are optimized for additive manufacturing, studies demonstrate that the process parameters have a major influence on the mechanical and quality characteristics, and to obtain the ideal characteristics, it is necessary to optimize the process parameters [6,7,8,9,10]. The extraordinary potential of additive manufacturing technologies and the interest in their adoption in leading industries have given rise to research directions aimed at studying and improving the mechanical characteristics of parts, integrating recycled materials into additive manufacturing, and studying and optimizing parameters to achieve dimensional requirements [11,12,13,14]. In the study presented in [15], the influence of the following process parameters is highlighted: the height of the deposited layer in one pass (0.10–0.20 mm), the printing speed (40–120 mm/s), and the filling density (10–50%). In the study presented in [16], the influence of the filling pattern (Triangular, Rectilinear, Gyroid, Honeycomb, and Concentric) on the parts manufactured additively from recycled PLA is investigated. In the study presented in [17], the influence of the platform temperature was investigated, with the temperature varying in the range of 60–80 °C. The study by the authors of [18] focuses on investigating the influence of thermoplastic extrusion parameters (printing speed, extrusion temperature, and platform temperature) on the characteristics of parts manufactured from PLA, ABS, PETG, PA6, and PP. The study presented in [19] highlights the influence of the extrusion temperature and the printing profile on the mechanical characteristics. The study presented in [20] aims to investigate the influence of thermoplastic extrusion parameters (layer height deposited in one pass, extrusion temperature, and part orientation) on the dimensional accuracy of additively manufactured parts. In [21], the influence of thermoplastic extrusion parameters (printing speed, filling percentage, and extrusion temperature) on dimensional accuracy is evaluated. In [22], the impact of thermoplastic extrusion parameters (layer height deposited in one pass, printing speed, and extrusion temperature) on roughness and dimensional characteristics is highlighted. The study presented in [23] focuses on evaluating the influence of thermoplastic extrusion parameters (layer height deposited in one pass, material type, printing speed, and nozzle diameter) on dimensional deviations. The dimensional characteristics of the parts made by additive technologies must comply with the rigorous standards of leading industries such as the medical industry, automotive industry, aerospace industry, and defense industry. This is the subject of the works presented in [24,25,26,27,28,29,30,31,32,33,34,35,36], in which possibilities for improving the dimensional accuracy of parts manufactured by additive thermoplastic extrusion are investigated. In the works presented in [24,25,26,27,28,29], the influence of printing time, temperature, and productivity on the roughness and dimensions of parts manufactured by additive thermoplastic extrusion is investigated. In the works presented in [30,31,32,33,34,35,36], ways of evaluating, optimizing, and predicting the impact of additive manufacturing parameters on dimensional accuracy are studied. In this sense, for the purpose of this study, a bibliographic study was conducted to understand the current state of research on the dimensional accuracy of parts manufactured by thermoplastic extrusion, the materials used, and the problems discovered. The research presented in [37] studies the mechanical characteristics of parts manufactured by additive manufacturing of polyethylene terephthalate glycol (PETG). Given that traditional tensile testing methods cannot consider the structural heterogeneity and anisotropy of 3D-printed materials, the authors suggest an improved methodology. This methodology is based on an inverse problem-solving method that combines experimental deformation measurements by Digital Image Correlation (DIC) with numerical simulations of the finite element method (FEM). By gradually reducing the differences between the experimental and simulated data, the study precisely establishes the main parameters of the material (Young’s modulus and Poisson’s ratio).

In the article presented in [38], the authors present a systematic analysis of the optimization of the parameters of the AM process by thermoplastic extrusion and their influence on the characteristics of the parts. The conclusions of the study certify that the optimization of the process parameters is of major importance for the quality of the parts, but also for the efficiency of the use of the equipment. In the paper presented in [39], the results of the research on the influence of the extrusion temperature on the dimensional characteristics of the parts manufactured by the thermoplastic extrusion of polylactic acid (PLA) and acrylonitrile butadiene styrene (ABS) are presented. The conclusions of the study show that the average dimensional accuracy of the samples manufactured from polylactic acid (PLA) was better than the average dimensional accuracy of the samples manufactured from acrylonitrile butadiene styrene (ABS). The increase in the extrusion temperature caused an increase in the average width of the strip manufactured from PLA, with a magnitude of deviation ranging from ±0.010 mm to ±0.034 mm. In [40], the effects of variable process parameters on the dimensional accuracy of parts manufactured by thermoplastic extrusion using the response surface methodology are studied. The following process parameters were used for the study: layer height deposited in one pass (0.12, 0.20, and 0.25 mm), filling pattern (linear, hexagonal, and star), raster angle (0, 45, and 90°), and filling density (40, 60, and 80%). The smallest deviations of linear and radial dimensions were obtained for the fabricated specimens using the layer thickness deposited in one pass of 0.15 mm, linear filling pattern, raster angle 90°, and filling density of 40%.

In [41], the authors present the results of a comprehensive study on the dimensional optimization of 3D FFF parts made of acrylonitrile butadiene styrene (ABS), polylactic acid (PLA), high impact polystyrene (HIPS), and polyethylene terephthalate glycol (PETG), as a solution for a correct design. The results of the study show that the factors that can cause dimensional variations in parts manufactured by thermoplastic extrusion are: thermal expansion of the material, faulty printer calibration, cooling time, mechanical stress, environmental conditions, and design accuracy. At the same time, the study emphasizes the importance of dimensional differences that can be corrected by adjusting the printing dimensions.

In [42], the influence of printing speed and filament color on the dimensional accuracy and tensile properties of samples manufactured by thermoplastic extrusion of polylactic acid (PLA) is studied. Regarding dimensional accuracy, the results of the study show that despite the increase in printing speed, the dimensional deviations of width and thickness remained within acceptable ranges. Among the speeds tested, the speed of 300 mm/s generated the best dimensional accuracy, with the cross-sectional deviations being minimal for both colors of the material (black and natural). At speeds above 200 mm/s, black PLA stood out for its superior dimensional accuracy compared to natural PLA.

In the paper presented in [43], a theoretical–experimental research was conducted on the influence of the layer height deposited in one pass and the filling percentage on the dimensional accuracy of tensile specimens additively manufactured from PLA. The results of the study show that the highest dimensional accuracy was obtained for the measurement points corresponding to the thickness of the specimen. At the same time, according to the authors’ findings, increasing the layer height deposited in one pass generates improvements in the dimensional accuracy on the Y axis, and increasing the filling percentage significantly influences the dimensional accuracy on the Z axis.

Considering the current state of research on the dimensional accuracy of parts manufactured by thermoplastic extrusion, but also the need to integrate recycled materials in the field of thermoplastic extrusion additive manufacturing, it was concluded that certain directions of study have not yet been exploited. Research focusing on comparing the characteristics of virgin and recycled PETG/ASA is limited. In this regard, the authors considered it appropriate to carry out a comparative study on the influence of thermoplastic extrusion parameters, the height of the layer deposited in one pass, L_h_ = (0.10, 0.15, and 0.20) mm, and the filling percentage, I_d_ = (50, 75, and 100)%, on the dimensional accuracy of three-point bending specimens manufactured additively from virgin materials and recycled materials (r) from polyethylene terephthalate glycol (PETG) and acrylonitrile styrene acrylate (ASA). As requirements for reducing the amount of plastic waste increase, finding viable solutions for the use of recycled plastics represents a challenge and a priority.

To highlight the relevance of the analyzed bibliographic sources, using the VOS viewer version 1.6.20 software [44], a map was created regarding the co-occurrence of keywords in the specialized literature studied.

**Figure 1:**
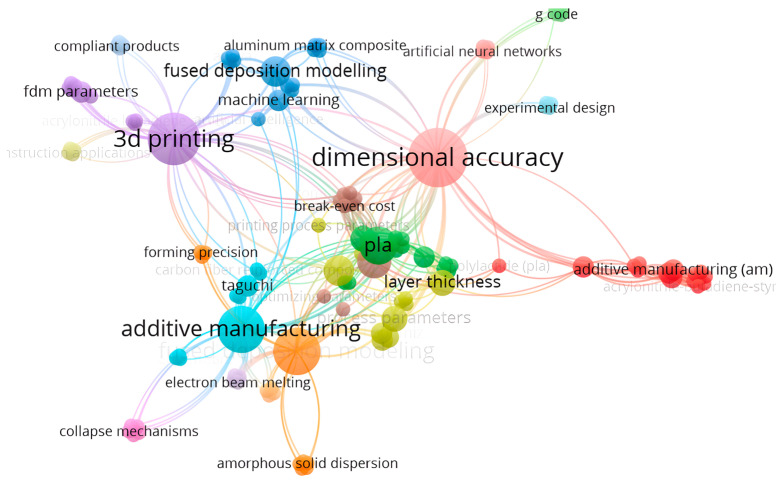
The network regarding the co-occurrence of keywords within the studied works.

Analyzing Figure 1, we can see several nodes that represent keywords; the size of the nodes is proportional to their frequency of occurrence. Links indicate the connection between nodes. Nodes are grouped by colors that correspond to a major objective in the research.

## 2. Additive Manufacturing by Thermoplastic Extrusion of PETG, rPETG, ASA, and rASA of Three-Point Bending Specimens and Determination of Dimensional Characteristics

**Figure 2:**
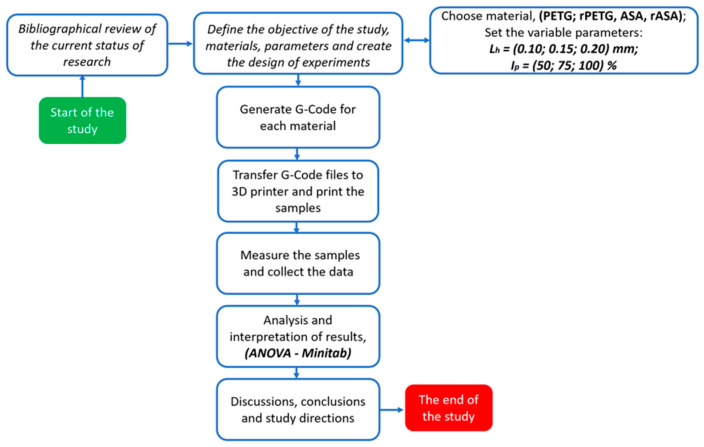
The working methodology of the study.

The study followed the workflow shown in Figure 2.

### 2.1. Additive Manufacturing of Specimens

To carry out the study, a total of 180 3-point bending specimens (45 PETG, 45 rPETG, 45 ASA, 45 rASA) were additively manufactured by thermoplastic extrusion on the QIDI Q1 Pro 3D printer using the parameters in Table 1 [45,46,47].

**Figure 3:**
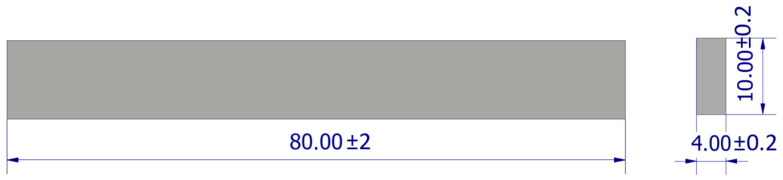
Shape and dimensions of the 3-point bending specimen.

The shape and dimensions of the 3-point bending specimen model are shown in Figure 3.

Table 1 shows the parameters used for additive manufacturing of 3-point bending specimens by thermoplastic extrusion of PETG, rPETG, ASA, and rASA filaments. All 4 filament types are from the Everfil brand and have a diameter of 1.75 mm. The printing parameters were chosen according to the filament manufacturer’s instructions, the slicer’s instructions, and previous studies on the same materials.

### 2.2. Determination of Dimensional Characteristics of 3-Point Bending Specimens Additively Manufactured by Thermoplastic Extrusion of PETG, rPETG, ASA, and rASA Filaments

**Figure 4:**
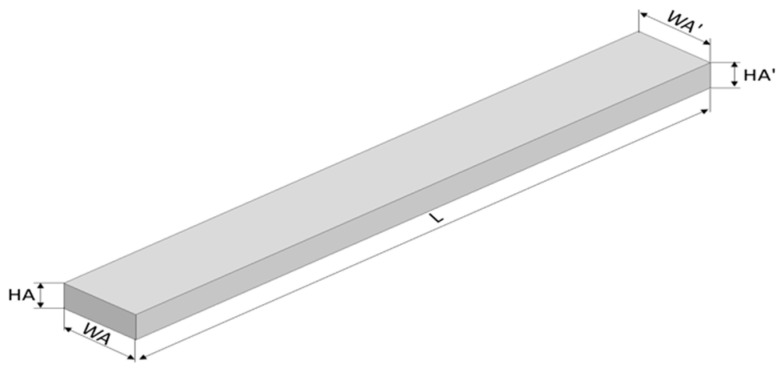
Measurement points of 3-point bending specimens.

To determine the dimensional characteristics of 3-point bending specimens additively fabricated by thermoplastic extrusion of PETG, rPETG, ASA, and rASA, the measurement points were defined according to Figure 4.

**Figure 5:**
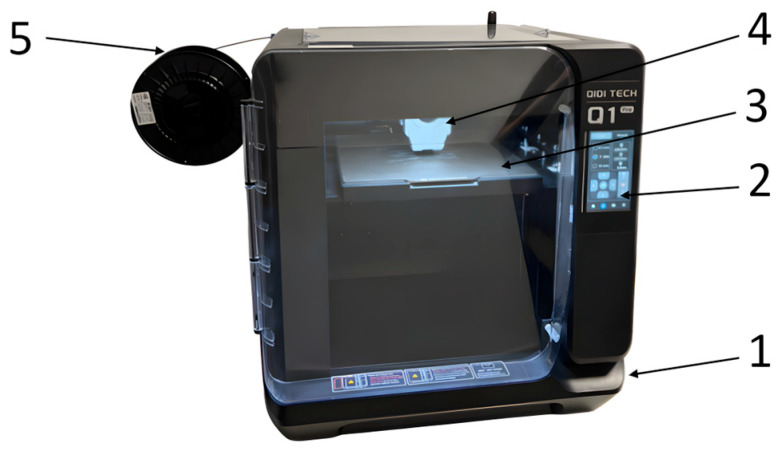
QIDI Q1 Pro 3D printer: 1—frame; 2—touchscreen command panel; 3—platform; 4—extruder; and 5—filament spool.

According to Figure 4 and Figure 5, dimensional characteristics were defined and determined by measurement, these are: L—length, WA—width A, HA—height A, WA’—width A’, and HA’—height A’.

Figure 5 shows the QIDI Q1 Pro 3D printer used to manufacture the 180 specimens.

**Figure 6:**
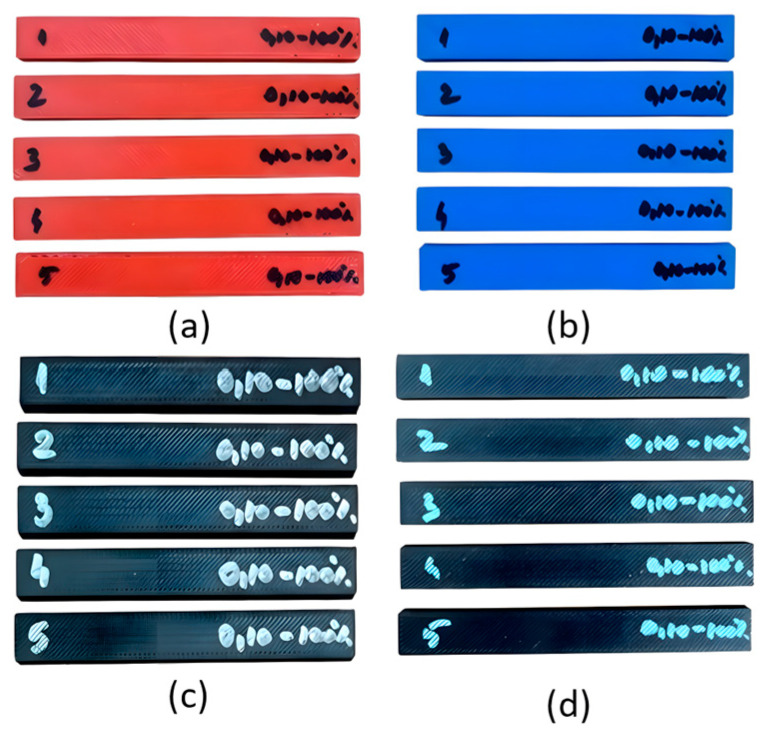
3D-printed 3-point bending specimens: (**a**) PETG; (**b**) ASA; (**c**) rPETG; and (**d**) rASA.

Figure 6 shows 20 3-point bending specimens (5 for each type of material).

Using the DeMeet 400 coordinate measuring machine, the dimensional characteristics of 3-point bending specimens manufactured by thermoplastic extrusion of PETG, rPETG, ASA, and rASA filaments were determined. For each specimen, the 5-dimensional characteristics were determined, ultimately resulting in 900 dimensional determinations, 180 determinations for each dimensional characteristic.

**Figure 7:**
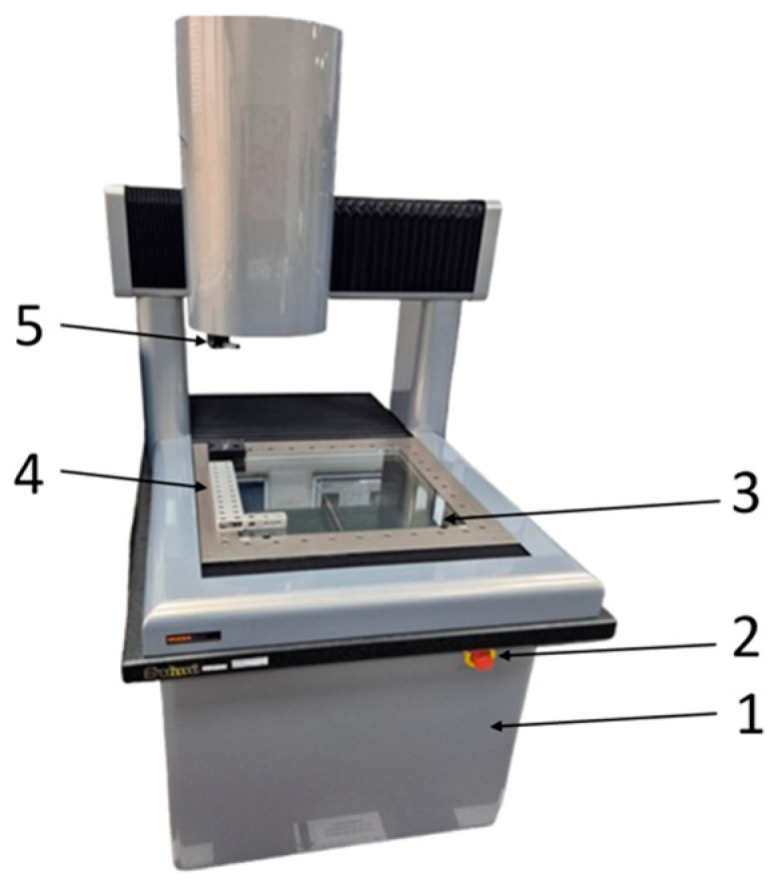
Coordinate measuring machine DeMeet 400: 1—frame; 2—emergency stop button; 3—support table; 4—base elements; and 5—rotary RDS + stylus.

Figure 7 shows the DeMeet 400 (Groningen, The Netherlands) coordinate measuring machine, which was used to perform the dimensional determinations in this study. The DeMeet 400 is a 3D laser coordinate measuring machine. It is used to determine the geometric dimensions (length, width, height, diameter, and depth) of a part on the 3 coordinate axes: X, Y, and Z.

**Figure 8:**
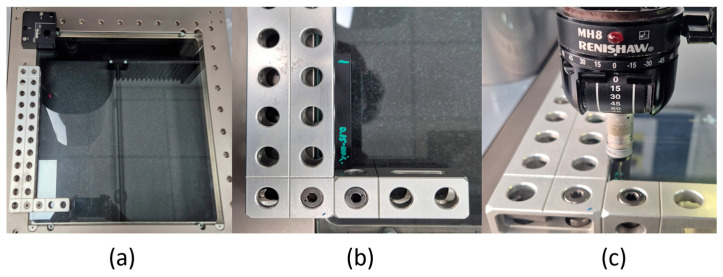
Methodology for measuring 3-point bending specimens on DeMeet 400: (**a**) creation of the basing system; (**b**) basing and fixing the specimen; and (**c**) measurement of the specimen.

After each stop, the machine automatically calibrates itself on the 3 axes (X, Y, and Z). The calibration process is completed when the machine is in the home position. Regarding the measurement method, a basing and fixing system was developed, and subsequently, to optimize the measurement process, a measurement program was developed. Figure 8 shows the steps taken to determine the dimensional characteristics of the 3-point bending specimens.

## 3. Results and Discussion

Using the values obtained from the 900-dimensional determinations on the Demeet 400 machine (see Figure 7), according to the measurement coordinates presented in Figure 4, the following statistical indicators were determined: arithmetic mean (1), dispersion (2), and standard deviation (3) [48].

(1) $$\bar{x}=\frac{\sum_{i=1}^{n}x_{i}}{n}$$

(2) $$\sigma^{2}=\frac{\sum_{i=1}^{n}{\left(x_{i}-\bar{x}\right)}^{2}}{n-1}$$

(3) $$\sigma =\sqrt{\frac{\sum_{i=1}^{n}{\left(x_{i}-\bar{x}\right)}^{2}}{n-1}}$$

The relation (1) corresponding to the arithmetic mean is used to determine the central value for each type of dimensional characteristic. It is calculated by summing all individual values ($x_{i}$) and dividing by their number (n).

The relation (2) corresponds to the dispersion, which quantifies the degree of dispersion of the measured values from their mean.

The relation (3) represents the root mean square deviation, which indicates the precision and reproducibility.

**Figure 9:**
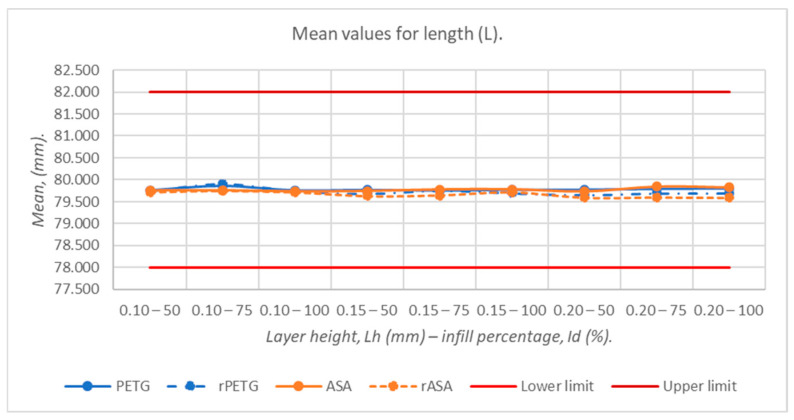
Average values of the lengths of 3-point bending specimens additively manufactured from PETG, rPETG, ASA, and rASA.

**Figure 10:**
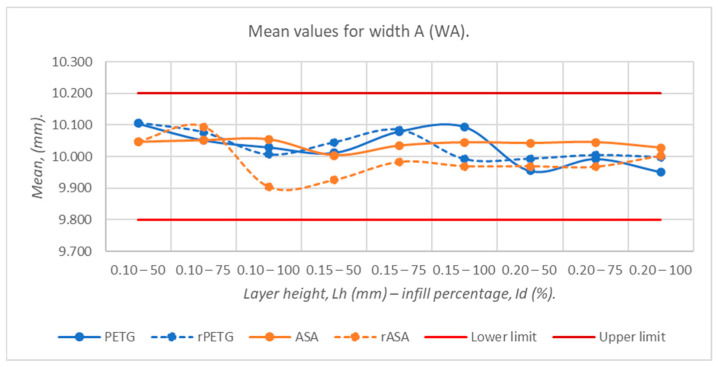
Average values of widths at point A (WA), corresponding to 3-point bending specimens additively manufactured from PETG, rPETG, ASA, and rASA.

**Figure 11:**
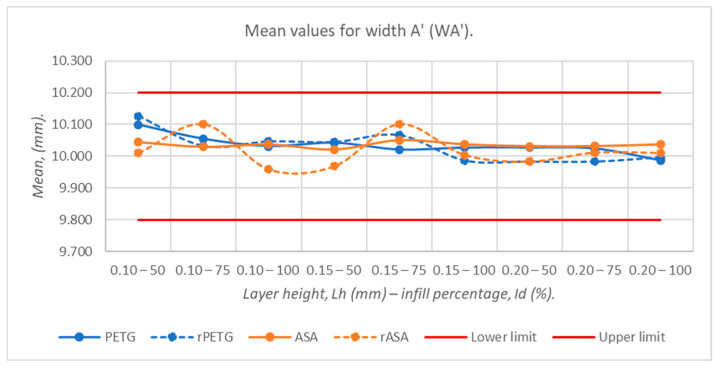
Average values of widths at point A’ (WA’), corresponding to 3-point bending specimens additively manufactured from PETG, rPETG, ASA, and rASA.

**Figure 12:**
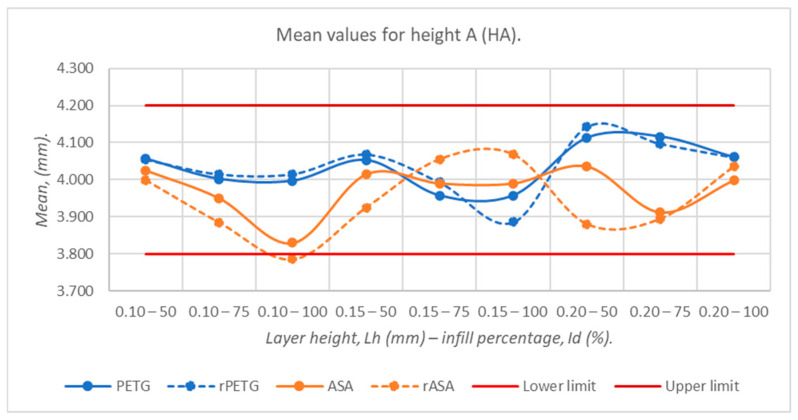
Average values of heights at point A (HA), corresponding to 3-point bending specimens additively manufactured from PETG, rPETG, ASA, and rASA.

**Figure 13:**
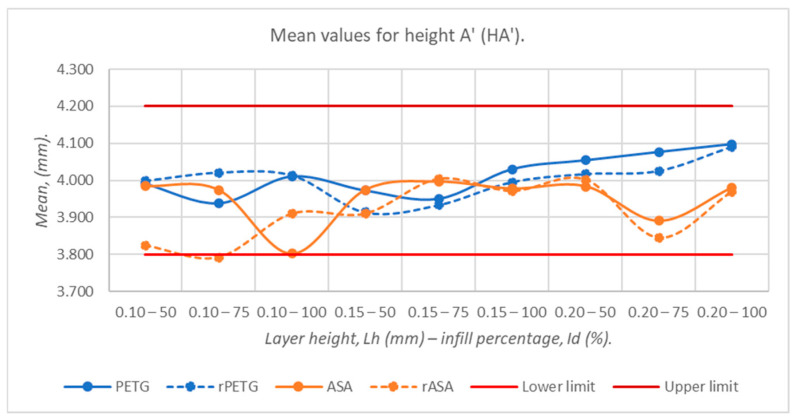
Average values of heights at point A’ (HA’), corresponding to 3-point bending specimens additively manufactured from PETG, rPETG, ASA, and rASA.

Figure 9, Figure 10, Figure 11, Figure 12 and Figure 13 graphically represent the arithmetic mean values corresponding to the dimensions of three-point bending specimens printed using the parameters presented in Table 1 by thermoplastic extrusion of PETG, rPETG, ASA, and rASA filaments.

Figure 9 shows that increasing layer height generally reduces dimensional accuracy, particularly for rASA and rPETG. In the case of the specimens fabricated from rASA, a significant thermal shrinkage was observed, being on average 0.34 mm compared to the nominal value. The minimum and maximum average values are located in the range of 79.589–79.910 mm and were recorded for the specimens additively manufactured from rASA filament, with L_h_ = 0.20 mm and I_d_ = 100%, and rPETG filament, with L_h_ = 0.10 mm and I_d_ = 75%, respectively. In the case of all sets of specimens, thermal shrinkage was observed (0.11–0.51%) by comparison with the nominal value.

Figure 10 shows the influence of the thermoplastic extrusion parameters, L_h_ = (0.10, 0.15, and 0.20) mm and I_d_ = (50, 75, and 100)%, as well as virgin and recycled PETG and ASA filaments on the average values of the widths at point A (WA), corresponding to the 3D-printed three-point bending specimens. The minimum average value (9.904 mm) corresponded to the pieces that were made from rASA with L_h_ = 0.10 mm and I_d_ = 100%, while the maximum average value (10.106 mm) is associated with rPETG filament pieces with L_h_ = 0.10 mm and I_d_ = 50%. For this dimension (WA), we observed the appearance of the over-sizing effect, which is influenced by the height of the layer deposited in one pass (L_h_). For PETG, rPETG, and ASA, good dimensional stability was observed with deviations of up to 0.039 mm from the nominal size, while for rASA, severe fluctuations (0.191 mm) and deviations of up to 0.15 mm from the nominal size were observed. For L_h_ = 0.20 mm, the average values tend towards the nominal value for all materials, this being due to the reduction in the number of layers, and therefore of the cumulative errors.

Figure 11 graphically represents the average values of the widths at point A’ (WA’), corresponding to the three-point bending specimens fabricated from PETG, rPETG, ASA, and rASA with L_h_ = (0.10, 0.15, and 0.20) mm and I_d_ = (50, 75, and 100)%. The minimum and maximum average values are located in the range of 9.958–10.126 mm; these were recorded for the specimens additively manufactured from rASA filament, with L_h_ = 0.10 mm and I_d_ = 100%, and rPETG filament, with L_h_ = 0.10 mm and I_d_ = 50%, respectively. Increasing L_h_ leads to dimensions close to the nominal ones. In the case of specimens manufactured with L_h_ = 0.10/0.15/0.20 mm, the average deviations from the nominal dimension are 0.47/0.31/0.09%.

Figure 12 shows the graphical representation of the average values of the heights at point A (HA), corresponding to the three-point bending specimens 3D-printed from virgin and recycled materials of PETG and ASA. The only material that exceeded the tolerance range is rASA with L_h_ = 0.10 mm and I_d_ = 100%. In the case of HA dimensions, increasing the height of the deposited layer at a transition from 0.10 mm to 0.15 mm leads to an increase in the heights of the specimens by 0.028 mm, and increasing L_h_ from 0.15 mm to 0.20 mm generates an increase in the heights of the specimens by 0.032 mm. The average values of the heights of the three-point bending specimens are in the range of 3.786–4.142 mm, with these values being obtained for the specimens additively manufactured by thermoplastic extrusion of the rASA filament using L_h_ = 0.10 mm and I_d_ = 100%, and rPETG using L_h_ = 0.20 mm and I_d_ = 50%, respectively. In the case of samples manufactured from PETG/rPETG, a pronounced over-dimensioning was observed, generated by the increase in the height of the deposited layer at a transition to 0.20 mm, and in the case of samples manufactured additively from ASA/rASA, the pronounced shrinkage was observed for L_h_ = 0.10 mm.

Figure 13 shows that increasing the height of the deposited layer in one pass (L_h_) from 0.10 mm to 0.15 mm generates an increase in the average heights of the three-point bending specimens by 0.031 mm, and increasing L_h_ from 0.10 mm to 0.15 mm and 0.20 mm increases the average heights of the three-point bending specimens by 0.065 mm. It is worth noting that the average heights of the specimens manufactured from rASA with L_h_ = 0.10 mm and I_d_ = 75% exceed the tolerance interval of 0.008 mm. The minimum average value (3.792 mm) corresponded to the pieces that were made from rASA with L_h_ = 0.10 mm and I_d_ = 75%, while the maximum average value (4.097 mm) is associated with PETG filament pieces with L_h_ = 0.20 mm and I_d_ = 100%. The average height of the 225 specimens is 3.970 mm, with the deviation from the nominal value being 0.030 mm.

**Figure 14:**
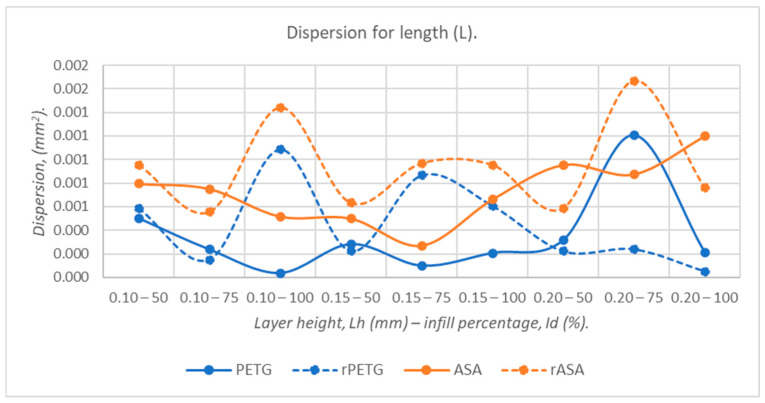
The dispersion values corresponding to the lengths (L) of 3-point bending specimens additively manufactured from PETG, rPETG, ASA, and rASA.

**Figure 15:**
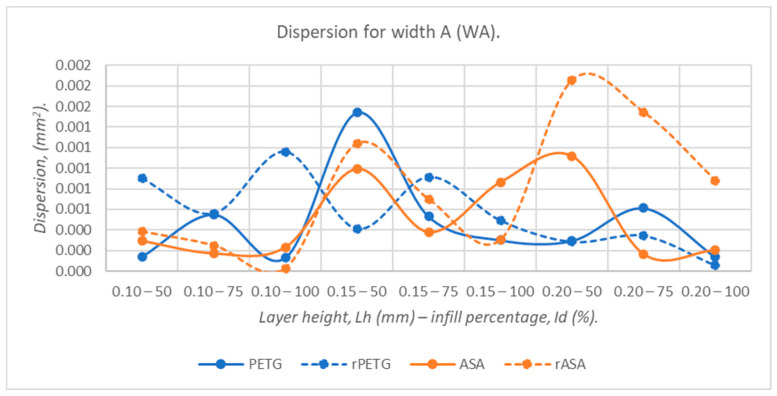
The dispersion values corresponding to the widths at point A (WA) of 3-point bending specimens additively manufactured from PETG, rPETG, ASA, and rASA.

**Figure 16:**
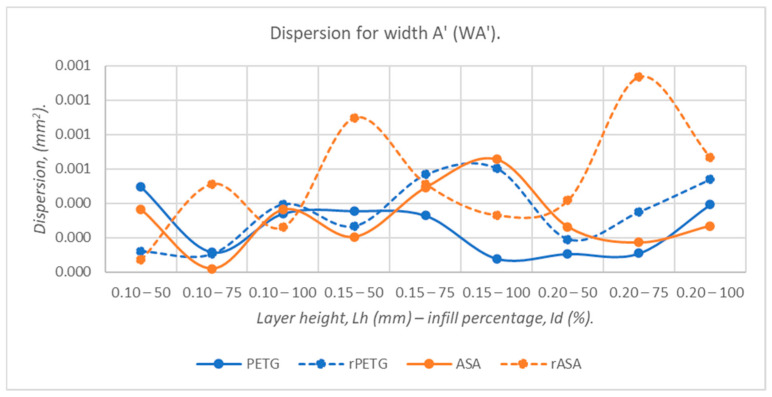
The dispersion values corresponding to the widths at point A’ (WA’) of 3-point bending specimens additively manufactured from PETG, rPETG, ASA, and rASA.

**Figure 17:**
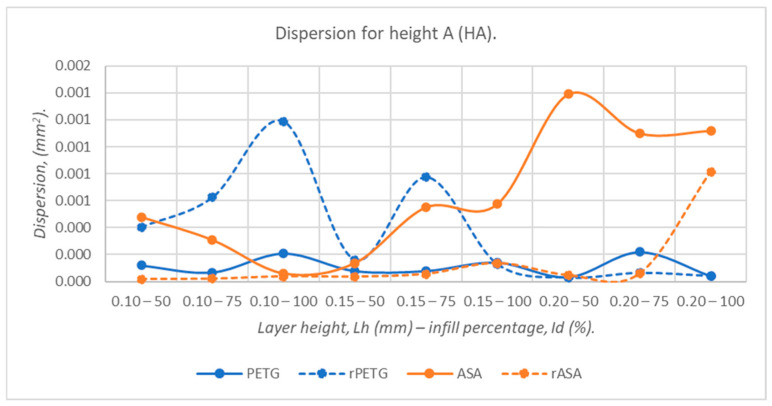
The dispersion values corresponding to the heights at point A (HA) of 3-point bending specimens additively manufactured from PETG, rPETG, ASA, and rASA.

**Figure 18:**
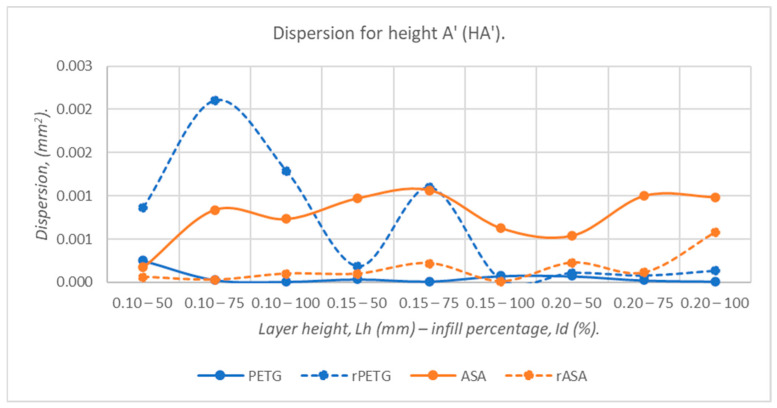
The dispersion values corresponding to the heights at point A’ (HA’) of 3-point bending specimens additively manufactured from PETG, rPETG, ASA, and rASA.

Figure 14, Figure 15, Figure 16, Figure 17 and Figure 18 graphically represent the dispersion values corresponding to the dimensions of three-point bending specimens 3D-printed using the parameters presented in Table 1 by thermoplastic extrusion of PETG, rPETG, ASA, and rASA filaments.

According to the graph presented in Figure 14, for lengths (L), the dispersions of virgin materials (PETG and ASA) are up to 23.50% lower than those of recycled materials (rPETG and rASA).

The data presented in Figure 15 show that the dispersions corresponding to the widths at point A (WA) of the three-point bending specimens of virgin materials (PETG and ASA) are up to 36.67% lower than those of recycled materials (rPETG and rASA).

Analyzing the graph in Figure 16, we observe that the dispersions corresponding to the widths at point A’ (WA’) of the three-point bending specimens 3D-printed from PETG and ASA offer superior dimensional stability (17.17–38.67%) compared to that of recycled materials (rPETG and rASA). The dispersions of virgin materials are lower by up to 41.62% compared to those of recycled materials.

Analyzing the dispersion curves in Figure 17 showing the influence of the thermoplastic extrusion parameters, L_h_ = (0.10, 0.15, and 0.20) mm and I_d_ = (50, 75, and 100)%, and of virgin (PETG and ASA) and recycled (rPETG, rASA) materials on the heights at point A (HA) of the three-point bending specimens, we observe that the materials that offer the best dimensional stability are PETG and rASA.

Analyzing the data presented in Figure 18, it can be concluded that the lowest dispersion values were recorded for PETG, which were 91.01% lower than those obtained for rPETG, 92.37% lower than those obtained for ASA, and 63.20% lower than those obtained for rASA.

**Figure 19:**
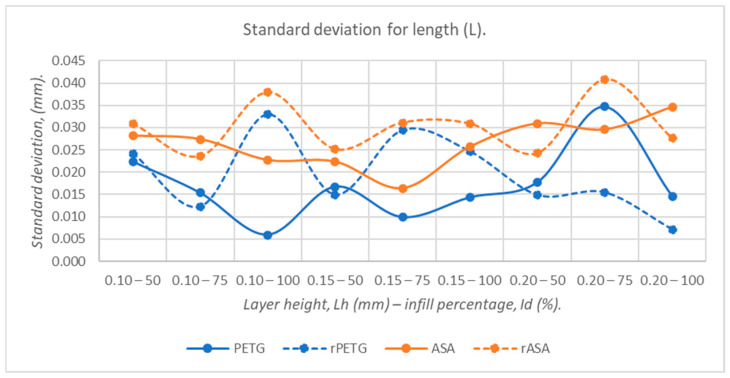
The root mean square deviations corresponding to the lengths of 3-point bending specimens additively manufactured from PETG, rPETG, ASA, and rASA.

**Figure 20:**
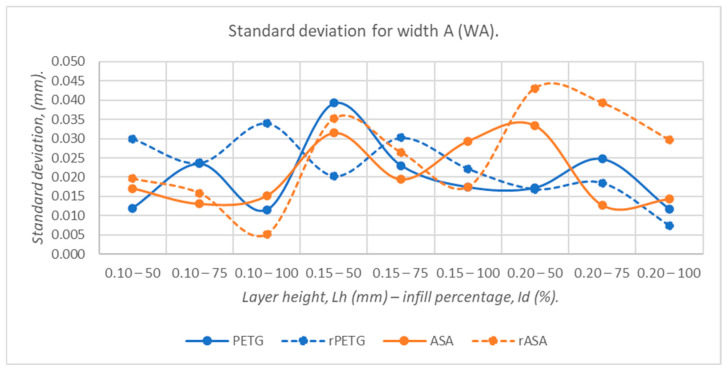
The root mean square deviations corresponding to the widths at point A (WA) of 3-point bending specimens additively manufactured from PETG, rPETG, ASA, and rASA.

**Figure 21:**
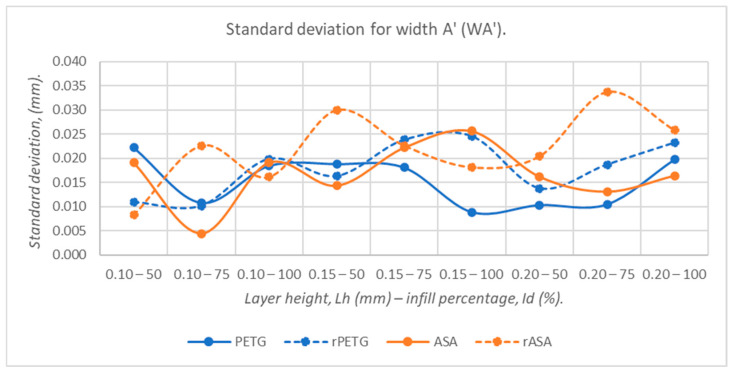
The root mean square deviations corresponding to the widths at point A’ (WA’) of 3-point bending specimens additively manufactured from PETG, rPETG, ASA, and rASA.

**Figure 22:**
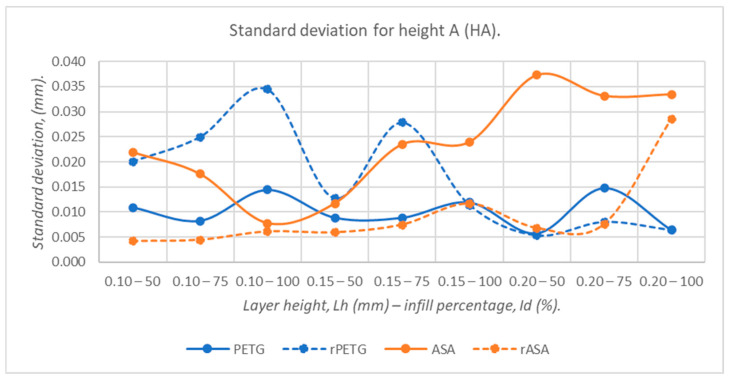
The root mean square deviations corresponding to the heights at point A (HA) of 3-point bending specimens additively manufactured from PETG, rPETG, ASA, and rASA.

**Figure 23:**
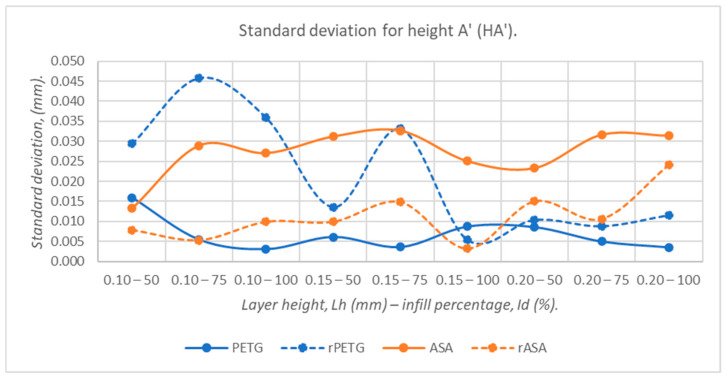
The root mean square deviations corresponding to the heights at point A’ (HA’) of 3-point bending specimens additively manufactured from PETG, rPETG, ASA, and rASA.

Figure 19, Figure 20, Figure 21, Figure 22 and Figure 23 graphically represent the values of the root mean square deviations corresponding to the dimensions of the three-point bending specimens manufactured additively using the parameters presented in Table 1 by thermoplastic extrusion of PETG, rPETG, ASA, and rASA filaments.

Analyzing Figure 19, we observe the significant influence of thermoplastic extrusion parameters (L_h_ and I_d_) and material type on the lengths of three-point bending specimens fabricated from PETG, rPETG, ASA, and rASA. Filaments manufactured from virgin materials (PETG and ASA) provide a significantly superior dimensional stability (12.62–13.70%) compared to filaments made from recycled materials (rPETG and rASA).

Analyzing Figure 20, we can see the influence of the thermoplastic extrusion parameters, L_h_ = (0.10, 0.15, and 0.20) mm and I_d_ = (50, 75, and 100)%, on the WA dimensions corresponding to the three-point bending specimens fabricated by thermoplastic extrusion of filaments from PETG, rPETG, ASA, and rASA. PETG, rPETG, and ASA are the materials for which low standard deviations were recorded, and for rASA, the highest values of standard deviations were recorded, which means high variability.

The graph presented in Figure 21 shows significant variations in the standard deviations corresponding to the WA’ dimension for the samples fabricated from rASA filament, with these being higher by 18.45–30.41% compared to those of PETG, rPETG, and ASA.

The graph shown in Figure 22 highlights the lack of capability of rPETG when using low layer heights of 0.10 and 0.15 mm and the low repeatability of ASA when increasing L_h_ from 0.10 mm to 0.15 mm and 0.20 mm. At the same time, high values of the standard deviation of rASA were observed when increasing the filling percentage (I_d_) at layer heights of (L_h_) of 0.20 mm. PETG is the material that offers the best repeatability in terms of specimen height.

According to the graph in Figure 23, PETG offers superior dimensional stability (69.14%) compared to recycled materials, rPETG, due to thermal stability.

After analyzing Figure 9, Figure 10, Figure 11, Figure 12, Figure 13, Figure 14, Figure 15, Figure 16, Figure 17, Figure 18, Figure 19, Figure 20, Figure 21, Figure 22 and Figure 23, it can be concluded that the thermoplastic extrusion parameters, L_h_ = (0.10, 0.15, and 0.20) mm and I_d_ = (50, 75, and 100)%, and the type of material (virgin and recycled) significantly influence the dimensional accuracy of parts manufactured additively by thermoplastic extrusion. Parts manufactured from recycled ASA tend to shrink when printed on large surfaces; this is the result of thermal degradation of the polymers.

## 4. Evaluation and Optimization of Thermoplastic Extrusion Parameters

Using the results of the mean square deviations determined with relation (3), the Minitab 20 statistical software [49], and the DoE function, graphs were drawn regarding the influence of the thermoplastic extrusion parameters, L_h_ = (0.10, 0.15, and 0.20) mm and I_d_ = (50, 75, and 100)%, and the material on the dimensional characteristics (L—length, WA—width A, HA—height A, WA—width A’, and HA’—height A’).

Pareto charts show the influence of the 3 factors, A = material, B = L_h_, and C = I_d_, on the studied dimensional characteristics. If the statistical value of the standardized effects of the factors is greater than or equal to 2.048, then the respective factors statistically influence the studied characteristic.

**Figure 24:**
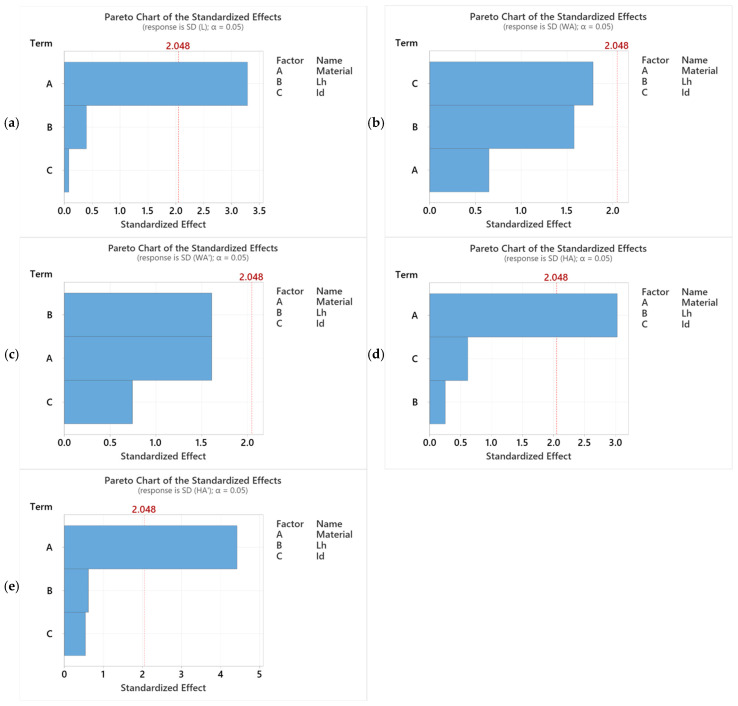
Pareto charts expressing the influence of the material (A = material) and the variable parameters of the thermoplastic extrusion (B = L_h_ and C = I_d_) on the mean square deviations for: (**a**) L—length; (**b**) WA—width A; (**c**) WA’—width A’; (**d**) HA—height A; and (**e**) HA’—height A’.

Analyzing Figure 24a, it can be seen that the lengths of the three-point bending specimens are significantly influenced by the type of material (factor A), and the secondary influence is represented by factor B—the height of the layer deposited in one pass (L_h_). According to Figure 24b, it can be concluded that the WA dimension is influenced by factor C, the filling percentage (I_d_), and by factor B, the height of the layer deposited in one pass (L_h_). The graph in Figure 24c shows that the WA’ dimension is significantly influenced by factor B, the height of the layer deposited in one pass, and by factor A, the material. The influence of factor C, the filling percentage, is 53.88% lower than the influence of factor A, the material. Analyzing Figure 24d,e, it can be seen that factor A, the material, decisively influences the dimensional accuracy on the measurement points HA—height A and HA’—height A’.

Based on the Pareto charts in Figure 24, the factors that influence the stability of the process were identified. In this sense, the analysis of variance (ANOVA) was performed to study whether these factors statistically influence the mean values of the studied characteristics.

**Figure 25:**
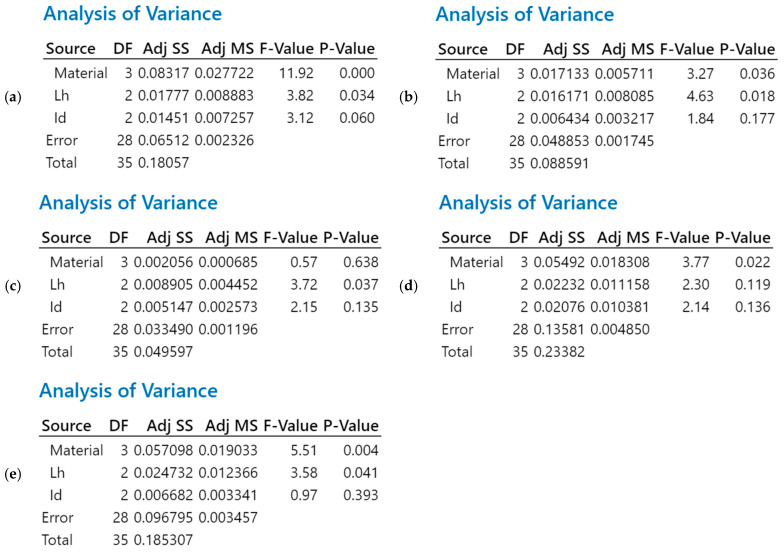
Results of the ANOVA analysis (General Linear Model) for: (**a**) L—length; (**b**) WA—width A; (**c**) WA’—width A’; (**d**) HA—height A; and (**e**) HA’—height A’.

Figure 25 presents the results of the ANOVA analysis for the five-dimensional characteristics studied.

Analyzing Figure 25a, we can see that the type of material categorically influences the average results of the lengths of the 3D-printed specimens from PETG, rPETG, ASA, and rASA (*p* < 0.05); in the same figure, we can see that the influence of the layer deposited in one pass (L_h_) is significant, but has a lower intensity than that of the type of material. The ANOVA analysis presented in Figure 25b shows that the height of the layer deposited in one pass is the parameter that significantly influences the average values of WA (*p* < 0.05), with the parameter that has a secondary influence being the type of material, and the filling percentage (I_d_) does not have a statistically significant influence. The results of the ANOVA analysis in Figure 25c show that the filling percentage has a very strong influence on the average values of the WA’ dimensions (*p* < 0.05), while the influence of the material type and the filling percentage is statistically insignificant. Figure 25d,e highlights the major influence of the material type on the average heights of the additively manufactured specimens from PETG, rPETG, ASA, and rASA, with this influence being confirmed by the ANOVA analysis (*p* < 0.05).

In order to identify the differences regarding the dimensional accuracy of each type of material, the analysis was performed using the Tukey grouping method. Table 2 summarizes the results of the analysis.

Analyzing the data in Table 2, we can conclude the following:

- For length (L), all materials undergo significant thermal shrinkage from −0.340 to −0.218 mm;
- PETG offers good dimensional accuracy, with the average results of the dimensions being located in the range of −0.21 to 0.035 mm from the nominal values, belonging to groups A and AB;
- rPETG stands out for its accuracy, similar to that of the virgin material; the average results of the studied characteristics were located in the range of −0.268 to 0.037 mm from the nominal values, with the material being included in groups A and AB;
- ASA offers good dimensional accuracy, with the average values of the dimensions having a deviation from −0.228 to 0.039 mm from the nominal values, with the material being found in groups A and AB;
- rASA suffered the greatest thermal shrinkage; the recycling process brought changes to the polymer structure. The average dimensional values are in the range of −0.340 to 0.016 mm compared to the nominal values, and the material was classified in 60% of cases in group B.

**Figure 26:**
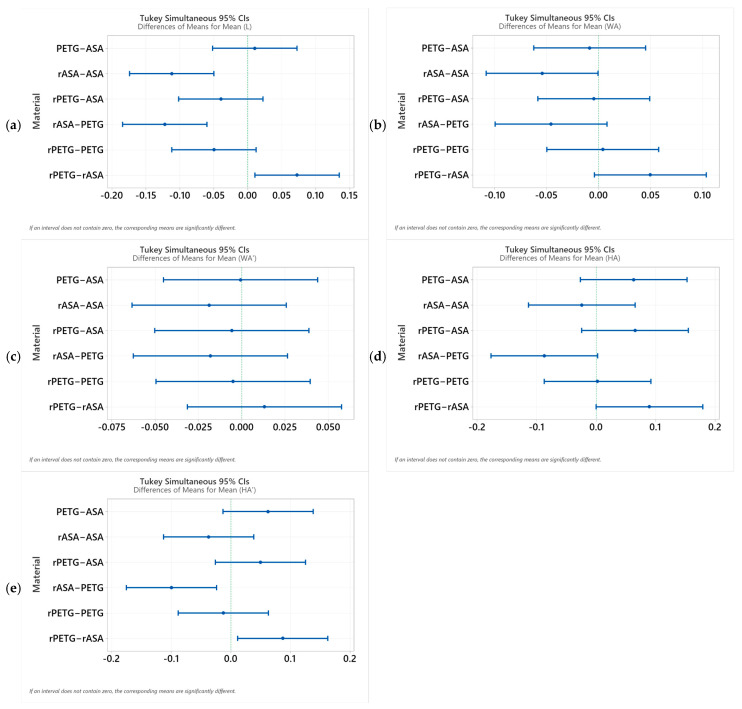
Tukey analysis results for: (**a**) L—length; (**b**) WA—width A; (**c**) WA’—width A’; (**d**) HA—height A; and (**e**) HA’—height A’.

To assess the statistical significance of the dimensional variations for the four studied materials (PETG, rPETG, ASA, and rASA), the post hoc Tukey HST test was applied, using a confidence level of 95%. The results obtained are summarized in Figure 26.

The graphs in Figure 26 express the confidence intervals for the differences in means. If the horizontal segments do not intersect the vertical line, then the difference between the two materials is statistically significant. Analyzing Figure 26a, it can be seen that the rASA material shows a significant decrease compared to rASA and rPETG. Figure 26b indicates a statistically significant variation for the rASA-ASA pair, and Figure 26c indicates that there are no statistically significant differences between the material combinations. Figure 26d,e shows that in the case of rASA-PETG and rPETG-rASA, there are statistically significant differences. Following the analysis of Figure 26, it can be concluded that rASA generates the largest variations compared to the other materials (PETG, rPETG, and ASA), indicating changes in the material properties after the recycling process.

**Figure 27:**
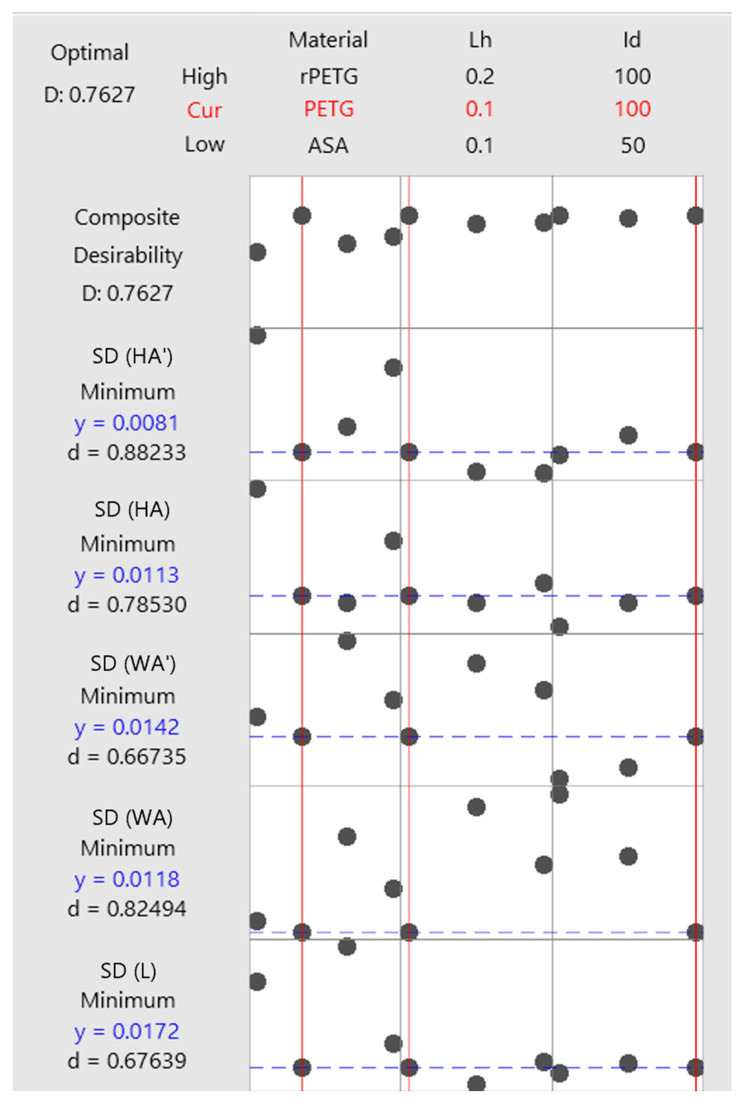
Graphs for optimizing thermoplastic extrusion parameters and material type to achieve ideal dimensional accuracy.

Using the root mean square deviations, the variable parameters of thermoplastic extrusion, L_h_ = (0.10, 0.15, and 0.20) mm and I_d_ = (50, 75, and 100)%, the four materials (PETG, rPETG, ASA, and rASA), and Minitab 20 software, graphs were generated to optimize the thermoplastic extrusion parameters and material type to achieve ideal dimensional accuracy (Figure 27).

The optimization graphs in Figure 27 indicate the following optimal settings:

- For rPETG, the optimal layer height deposited in one pass is L_h_ = 0.20 mm, and the filling percentage is I_d_ = 100%;
- For PETG, the optimal layer height deposited in one pass is L_h_ = 0.10 mm, and the filling percentage is I_d_ = 100%;
- For ASA, the optimal layer height deposited in one pass is L_h_ = 0.10 mm, and the filling percentage is I_d_ = 50%;
- rASA is not recommended for use in applications where dimensional accuracy is important.

## 5. Conclusions

This paper presents the results of the theoretical–experimental study on the evaluation and optimization of thermoplastic extrusion parameters to improve the dimensional accuracy of 3D-printed parts made of PETG, recycled PETG, ASA, and recycled ASA.

In the context of the transition to a circular economy and building a future based on sustainability, additive manufacturing technologies through plastic extrusion can absorb certain types of recycled plastics for the manufacture of parts. In this study, the influence of thermoplastic extrusion parameters (layer height deposited in one pass, L_h_, and filling percentage, I_d_) on the dimensional accuracy of three-point bending specimens additively manufactured from PETG, rPETG, ASA, and rASA was investigated. For the study, 180 three-point bending specimens (45 specimens for each material) were additively manufactured using L_h_ = (0.10, 0.15, and 0.20) mm and I_d_ = (50, 75, and 100)%. To determine the dimensions of the three-point bending specimens, five measurement points were defined for each specimen, and 900 measurements were performed using the DeMeet 400 coordinate measuring machine. The measurement results were used to determine the arithmetic means, dispersions, and standard deviations due to each combination of parameters and material.

Key findings:

- Dimensional accuracy is influenced by the thermoplastic extrusion parameters, L_h_ = (0.10, 0.15, and 0.20) mm and I_d_ = (50, 75, and 100)%, as well as the type of material used (see Figure 19, Figure 20, Figure 21, Figure 22 and Figure 23);
- The dimensional accuracy of samples made from virgin materials (PETG, ASA) is significantly higher than that of samples made from recycled materials (rPETG, rASA);
- Recycled materials (rPETG, rASA) suffer significant thermal contractions when deposited on large surfaces, due to thermal degradation of polymers;
- PETG is the material that offers the smallest dimensional variations;
- The mean square deviations of the coordinates L, WA, and WA’ of samples made from PETG are 13.12, 18.23, and 33.09% lower than the mean square deviations of samples made from rPETG, ASA, and rASA;
- The mean square deviations of the coordinates L, WA, and WA’ of the samples made of rPETG are 5.88 and 22.98% lower than the mean square deviations of the samples made of ASA and rASA;
- The mean square deviations of the coordinates HA and HA’ of the samples made of rASA are 46.81 and 59.63% lower than the mean square deviations of the samples made of rPETG and ASA, and 18.30% lower than those of the samples made of PETG.
- rASA is not suitable for the additive manufacturing of parts that require high precision (parts from the automotive or aerospace industry).

The optimal parameters that provide the lowest mean square deviations for each type of material are:

- L_h_ = 0.10–I_d_ = 100% for PETG;
- L_h_ = 0.10–I_d_ = 50% for rPETG;
- L_h_ = 0.10–I_d_ = 50% for ASA;
- L_h_ = 0.10–I_d_ = 50% for rASA.

This study demonstrates that recycled materials can be used for applications in the field of additive manufacturing technologies by thermoplastic extrusion for applications that do not require parts with high dimensional accuracy (consumer industry). Considering the results of this study demonstrating that rPETG has a dimensional accuracy similar to that of PETG and the results of the study presented by the authors of [45], where parts manufactured from rPETG using optimal parameters demonstrated superior mechanical properties to PETG, and the price per kilogram of rPETG is approximately 10% lower than that of PETG, we can conclude that recycling and reusing PETG is an optimal option from a technical and economic point of view. In order to have dimensional accuracy of parts manufactured additively from virgin materials, some compensation factors are required on the three coordinate axes (X, Y, and Z). In [46], the authors investigated the three-point bending properties of additively manufactured rASA specimens; the results of the study showed that for L_h_ = 0.10 and 0.15 mm, the bending strength of rASA specimens was 5.47–5.32% lower than that of ASA specimens using identical parameters, and in the case of specimens manufactured with L_h_ = 0.20 mm, rASA had results 5.63% higher. And in this case, the price of rASA filament is approximately 10% lower than that of virgin ASA filament.

Future research directions will be focused on studying the influence of filament color on dimensional accuracy, the influence of printing speed and extrusion temperature, and performing DSC and TGA analyses.

## Figures and Tables

**Table 1:** Extrusion parameters used for manufacturing 3-point bending specimens from PETG, rPETG, ASA, and rASA.

Constant Parameters	Variable Parameters
Orientation, O = XY Print speed, P_s_ = 120 mm/s Extrusion temperature, E_t_ = 250 °C PETG, rPETG platform temperature, P_t_ = 70 °C ASA, rASA platform temperature, P_t_ = 90 °C Infill pattern, I_p_ = Rectilinear	Layer height, L_h_ = (0.10, 0.15, and 0.20) mm Infill percentage, I_d_ = (50, 75, and 100)%
Plate adhesion, P_a_ = Brim	
Fill angle, F_a_ = 45° Pressure advance, P_a_ = 0.086 mm/s	

**Table 2:** Tukey test results for the dimensions of bending specimens made of PETG, ASA, rPETG, and rASA.

Dimensional Characteristic	Material	N	Mean	Grouping
L—length	PETG	9	79.782	A
	ASA	9	79.722	A
	rPETG	9	79.732	A
	rASA	9	79.660	B
WA—width A	ASA	9	10.039	A
	rPETG	9	10.343	AB
	PETG	9	10.304	AB
	rASA	9	9.984	B
WA’—width A’	ASA	9	10.035	A
	PETG	9	10.035	A
	rPETG	9	10.030	A
	rASA	9	10.017	A
HA—height A	rPETG	9	4.037	A
	PETG	9	4.035	A
	ASA	9	3.971	A
	rASA	9	3.947	A
HA’—height A’	PETG	9	4.013	A
	rPETG	9	4.000	A
	ASA	9	3.951	AB
	rASA	9	3.914	B

## Data Availability

Data are contained within the article. The data presented in this study are available upon request from the corresponding author.
